# Supplementary material for: F26BP enables control of glycolysis rate independent of energy state
Source: bioRxiv. 2026 Jan 31:2026.01.31.703051. Preprint. [Version 1] doi: 10.64898/2026.01.31.703051 (PMC12873801; doi:10.64898/2026.01.31.703051)
Supplement: 1 [file NIHPP2026.01.31.703051V1-supplement-1.pdf]

bioRxiv preprint doi: https://doi.org/10.64898/2026.01.31.703051; this version posted January 31, 2026. The copyright holder for this preprint (which was not certified by peer review) is the author/funder, who has granted bioRxiv a license to display the preprint in perpetuity. It is made available under aCC-BY 4.0 International license.

SUPPLEMENTAL TABLES

Table S1. Guide RNA Sequences used to generate *PFKFB1-4* knockout ($PFKFB_0$)

CRISPR-Cas9 sgRNA	Sequence (5'→ 3')
sgPFKFB1 #A	TTTAGGCCAGTATCGACGAG
sgPFKFB1 #B	CCAACACTACCAGAGAACGA
sgPFKFB2 #A	AATCATAACGATCAGAGTCG
sgPFKFB2 #B	TGTTAAGGCGTATCTCACTG
sgPFKFB3 #A	GCCCACCATGACGATGACGG
sgPFKFB3 #B	CGTCGGGGAGTATCGCCGGG
sgPFKFB4 #A	TGTAGGTCTTGACCACGTCC
sgPFKFB4 #B	GGAACCGCCGGACGTCACGG

Table S2. PCR and Sequencing Primers used to evaluate *PFKFB1-4* Insertion/Deletion & Intragenic Deletion Frequency

Target	Primer #1		Primer #2		Primer #3	
	Direction	Sequence (5'→ 3')	Direction	Sequence (5'→ 3')	Direction	Sequence (5'→ 3')
PFKFB1 Intragenic	Forward	GAAGTTTTAAAAGCACA TTTTGTAGCTA	Reverse	TTCCTCATGGCTGAGAT AGTTGTGA	Reverse	AGCTTATGGTTGCGG ATTTG
PFKFB1 Guide A	Forward	TGCAGGGCTGTTGTGA GGAATAAATA	Reverse	TGGGCAGGAAAATGGA AGGGCT		
PFKFB1 Guide B	Forward	GGGCCCAATATGTGCT TGTGAAAT	Reverse	GCACCCCTCCTTTCTTG TTCTGCT		
PFKFB2 Intragenic	Forward	GCCTGCCTGATATCTCC TTATTT	Reverse	AGGAGACAAGTCTGGG AATAGA	Reverse	TCATGTCCCTCCTCTC CCG
PFKFB2 Guide A	Forward	CTCCAAGCATTAGACCT TCCTT	Reverse	TGCTATTTCTGCCAAAA GCACCCT		
PFKFB2 Guide B	Forward	GGATGGTGTGAAAACC CTCAGT	Reverse	TCATGTCCCTCCTCTCC CG		
PFKFB3 Intragenic	Reverse	CATCTCTCAAGGCAGCT AAGG	Forward	GTGGATAGGCACTGGG TATTT	Forward	CCTCCCTGACCTACTT TCATTT
PFKFB3 Guide A	Forward	CCTCCCTGACCTACTTT CATTT	Reverse	CAGGATCAGACTCCCC GAC		
PFKFB3 Guide B	Forward	TGAGGGTAGCGTAGGA CTGG	Reverse	CATCTCTCAAGGCAGCT AAGG		
PFKFB4 Intragenic	Reverse	TGGAGTGAGTTGCCAG AAAG	Forward	AGAGAGGTTGGGAAGC TAGT	Forward	GGGCATGACGAAGAG AATAGAG
PFKFB4 Guide A	Forward	GGGCATGACGAAGAGA ATAGAG	Reverse	GGATGTGTGCCTATGA GGTATG		
PFKFB4 Guide B	Forward	GGAGCAGAGTTAGCCA AACACT	Reverse	TGGAGTGAGTTGCCAG AAAG		

bioRxiv preprint doi: https://doi.org/10.64898/2026.01.31.703051; this version posted January 31, 2026. The copyright holder for this preprint (which was not certified by peer review) is the author/funder, who has granted bioRxiv a license to display the preprint in perpetuity. It is made available under aCC-BY 4.0 International license.

Table S3. Metabolites utilized in PFKFB3 Regulator Screen

Chemical	Source	Cat#
Adenosine 5′-triphosphate disodium salt hydrate (ATP)	Millipore Sigma	A6419
Potassium Phosphate Monobasic (KH_2PO4)	Fisher Scientific	P285
Adenosine 5′-diphosphate sodium salt (ADP)	Millipore Sigma	A2754
Adenosine 5′-monophosphate monohydrate (AMP)	Millipore Sigma	A2252
D-Glucose 6-phosphate sodium salt (G6P)	Millipore Sigma	G7879
α-D-Glucose 1-phosphate disodium salt hydrate (G1P)	Millipore Sigma	G7000
D-Fructose-6-phosphate disodium salt (F6P)	Thermo Scientific	J66311.03
D-Fructose 1,6-bisphosphate trisodium salt hydrate (F16BP)	Millipore Sigma	F6803
Dihydroxyacetone phosphate dilithium salt (DHAP)	Millipore Sigma	D7137
DL-Glyceraldehyde 3-phosphate solution (GAP)	Millipore Sigma	G5251
D-(−)-3-Phosphoglyceric acid disodium salt (3PG)	Millipore Sigma	P8877
L-2-Phosphoglyceric acid disodium salt hydrate (2PG)	Millipore Sigma	19710
Phosphoenol-pyruvate (PEP)	Millipore Sigma	10108294001
Sodium Pyruvate	Thermo Scientific	11360070
Sodium L-Lactate	Millipore Sigma	71718
α-Ketoglutaric acid (α-KG)	Millipore Sigma	75890
D-α-Hydroxyglutaric acid sodium salt	Cayman Chemical	C832G02
Succinic acid	Thermo Scientific	219552500
Fumaric acid	Millipore Sigma	47910
Itaconic acid	Millipore Sigma	I29204
cis-Aconitic acid	Millipore Sigma	A3412
Sodium citrate tribasic dihydrate	Millipore Sigma	C8532
L-(−)-Malic acid	Millipore Sigma	02288
Acetyl coenzyme A lithium salt (Acetyl CoA)	Millipore Sigma	A2181
Oxaloacetic acid (OAA)	Millipore Sigma	O4126
Malonyl coenzyme A lithium salt (Malonyl CoA)	Millipore Sigma	M4263
Oleoyl coenzyme A lithium salt (Oleyl CoA)	Millipore Sigma	O1012
Palmitoyl coenzyme A lithium salt (Palmitoyl CoA)	Millipore Sigma	P9716
sn-Glycerol 3-phosphate lithium salt (G3P)	Millipore Sigma	P94124
L-Serine	Millipore Sigma	S4500
L-Alanine	Millipore Sigma	A7627
L-Glycine	Millipore Sigma	50046
L-Glutamine	Millipore Sigma	G8540
L-Leucine	Millipore Sigma	L8000
6-Phosphogluconic Acid Trisodium Salt Dihydrate	Millipore Sigma	P7877
D-Ribulose 5-phosphate sodium salt (Ribulose-5P)	Millipore Sigma	R9875
D-Ribose 5-phosphate disodium salt hydrate (Ribose-5P)	Millipore Sigma	R7750
D-Xylulose 5-phosphate lithium salt (Xylulose-5P)	Millipore Sigma	15732
D-Sedoheptulose 7-phosphate lithium salt (Sedoheptulose-7P)	Millipore Sigma	78832
D-Erythrose 4-phosphate sodium salt (Erythrose-4P)	Millipore Sigma	E0377
β-Nicotinamide adenine dinucleotide phosphate hydrate (NADP)	Millipore Sigma	N5755
β-Nicotinamide adenine dinucleotide 2′-phosphate reduced tetrasodium salt hydrate (NADPH)	Millipore Sigma	N6505
β-Nicotinamide adenine dinucleotide hydrate (NAD)	Millipore Sigma	N1636
β-Nicotinamide adenine dinucleotide, reduced disodium salt hydrate (NADH)	Millipore Sigma	N8129

bioRxiv preprint doi: https://doi.org/10.64898/2026.01.31.703051; this version posted January 31, 2026. The copyright holder for this preprint (which was not certified by peer review) is the author/funder, who has granted bioRxiv a license to display the preprint in perpetuity. It is made available under aCC-BY 4.0 International license.

Table S4. Cell Culture Media Composition

Component (mg/L)	Standard DMEM (Gibco #11995)	Minimal DMEM (US Biological Life Sciences #D9800-D28)
Glycine	30	0
L-Arginine hydrochloride	84	0
L-Cystine 2HCl	63	0
L-Glutamine	584	0
L-Histidine hydrochloride-H_2O	42	0
L-Isoleucine	105	0
L-Leucine	105	0
L-Lysine hydrochloride	146	0
L-Methionine	30	0
L-Phenylalanine	66	0
L-Serine	42	0
L-Threonine	95	0
L-Tryptophan	16	0
L-Tyrosine disodium salt dihydrate	104	0
L-Valine	94	0
Choline chloride	4	4
D-Calcium pantothenate	4	4
Folic Acid	4	4
Niacinamide	4	4
Pyridoxine hydrochloride	4	4
Riboflavin	0.4	0.4
Thiamine hydrochloride	4	4
i-Inositol	7.2	7.2
Calcium Chloride ($CaCl_2$) (anhyd.)	200	265
Ferric Nitrate ($Fe(NO_3)_3$-$9H_2O$)	0.1	0.1
Magnesium Sulfate (MgSO4) (anhyd.)	97.67	97.67
Potassium Chloride (KCl)	400	400
Sodium Bicarbonate ($NaHCO_3$)	3700	0
Sodium Chloride (NaCl)	6400	6400
Sodium Phosphate monobasic (NaH_2PO_4-H_2O)	125	109
D-Glucose (Dextrose)	4500	0
Phenol Red	15	15.9
Sodium Pyruvate	110	0

bioRxiv preprint doi: https://doi.org/10.64898/2026.01.31.703051; this version posted January 31, 2026. The copyright holder for this preprint (which was not certified by peer review) is the author/funder, who has granted bioRxiv a license to display the preprint in perpetuity. It is made available under aCC-BY 4.0 International license.

Table S5. Primary & Secondary Antibodies used for Western Blotting

Antibody	Source	Identifier
Rabbit polyclonal 6-phosphofructo-2-kinase/fructose-2,6-biphosphatase 3 (PFKFB3)	Proteintech	Cat#13763-1-AP; RRID: AB_2162854
Rabbit monoclonal 6-phosphofructo-2-kinase/fructose-2,6-biphosphatase 2 (PFKFB2)	Cell Signaling Technologies	Cat#13045; RRID: AB_2798097
Rabbit polyclonal DYKDDDDK (Flag) Tag	Cell Signaling Technologies	Cat#2368; RRID: AB_2217020
Rabbit polyclonal Firefly Luciferase	Thermo Fisher Scientific	Cat#PA5-32209; RRID: AB_2549682
Rabbit monoclonal β-Actin	Cell Signaling Technologies	Cat#4970; RRID: AB_2223172
Rabbit monoclonal Hexokinase 1 (HK1)	Cell Signaling Technologies	Cat#2024; RRID: AB_2116996
Rabbit monoclonal Hexokinase 2 (HK2)	Cell Signaling Technologies	Cat#2867; RRID: AB_2232946
Rabbit monoclonal Phosphofructokinase platelet isozyme (PFKP)	Cell Signaling Technologies	Cat#8164; RRID: AB_2713957
Rabbit monoclonal Glyceraldehyde-3-phosphate dehydrogenase (GAPDH)	Cell Signaling Technologies	Cat#2118; RRID: AB_561053
Rabbit monoclonal Pyruvate kinase M2 isoform (PKM2)	Cell Signaling Technologies	Cat#4053; RRID: AB_1904096
Rabbit monoclonal Mitochondrial pyruvate carrier 1 (MPC1)	Cell Signaling Technologies	Cat#14462; RRID: AB_2773729
Rabbit monoclonal Mitochondrial pyruvate carrier 2 (MPC2)	Cell Signaling Technologies	Cat#46141; RRID: AB_2799295
Rabbit monoclonal Citrate Synthase	Cell Signaling Technologies	Cat#14309; RRID: AB_2665545
Rabbit monoclonal Aconitase 2 (ACO2)	Cell Signaling Technologies	Cat#6571; RRID: AB_2797630
Rabbit polyclonal Isocitrate dehydrogenase 1 (IDH1)	Cell Signaling Technologies	Cat#3997
Rabbit monoclonal Isocitrate dehydrogenase 2 (IDH2)	Cell Signaling Technologies	Cat#56493; RRID: AB_2799511
Goat anti-rabbit IgG, HRP-linked antibody	Cell Signaling Technologies	Cat#7074; RRID: AB_2099233

bioRxiv preprint doi: https://doi.org/10.64898/2026.01.31.703051; this version posted January 31, 2026. The copyright holder for this preprint (which was not certified by peer review) is the author/funder, who has granted bioRxiv a license to display the preprint in perpetuity. It is made available under aCC-BY 4.0 International license.

SUPPLEMENTAL DATA

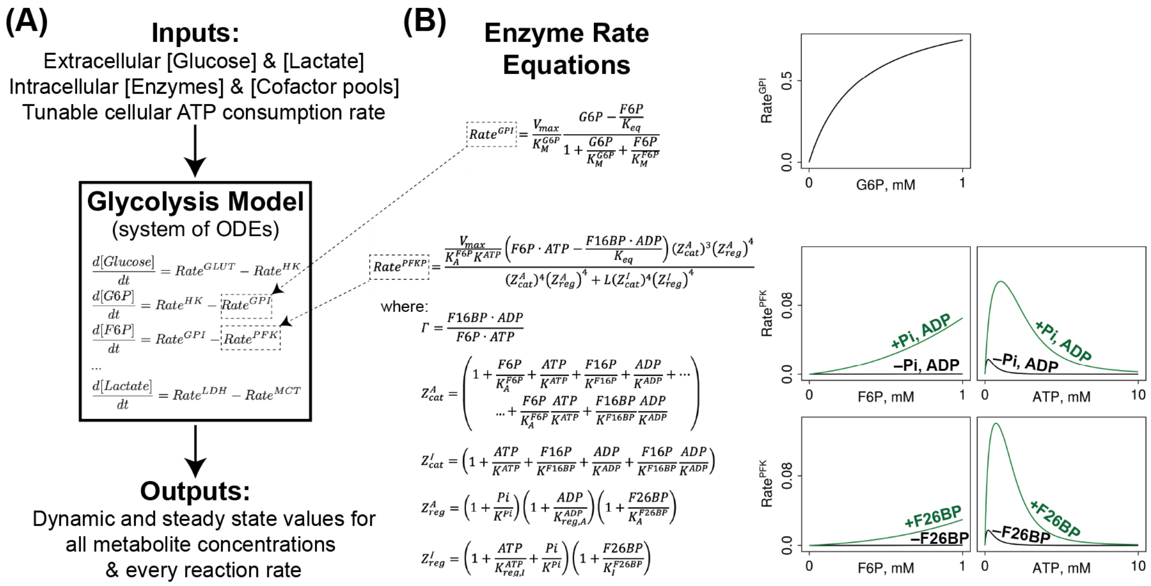

Figure S1. Biophysical model of mammalian glycolysis overview. (A) Schematic of the glycolysis inputs and outputs. (B) Kinetic rate equations are shown for GPI and PFK, with special attention to allosteric regulation by F26BP. Plots are actual GPI and PFK rates calculated by the respective equations with rates normalized by the V_{max}. Note the dramatic allosteric activation of the PFK rate in the presence of F26BP.[1]

bioRxiv preprint doi: https://doi.org/10.64898/2026.01.31.703051; this version posted January 31, 2026. The copyright holder for this preprint (which was not certified by peer review) is the author/funder, who has granted bioRxiv a license to display the preprint in perpetuity. It is made available under aCC-BY 4.0 International license.

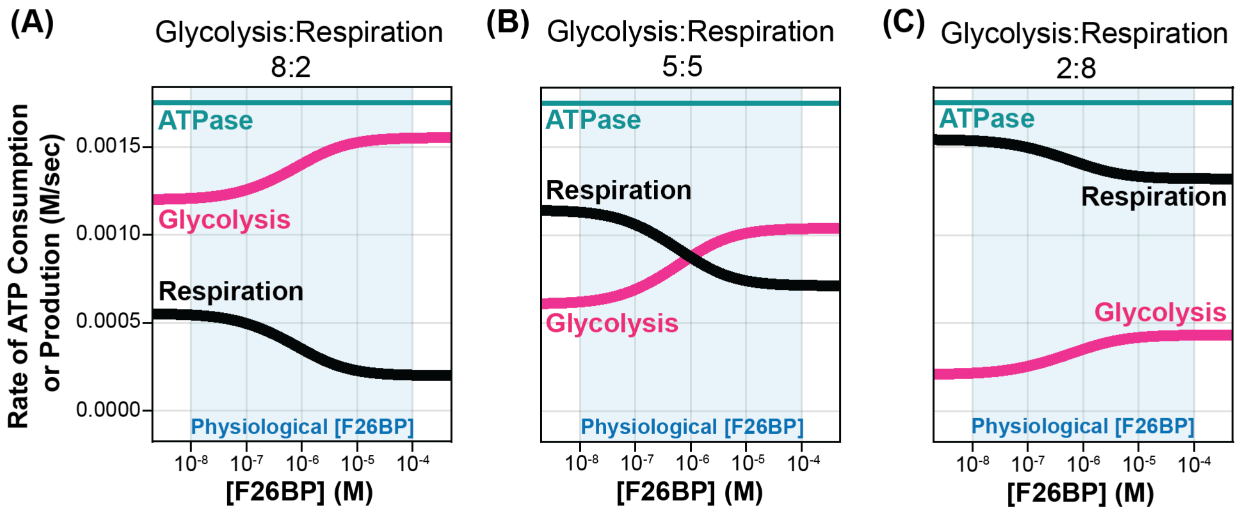

Figure S2. Effect of F26BP on glycolytic and respiratory ATP production rates at varying respiration-to-glycolysis ratios. Steady-state simulations of a glycolysis model with added ATP Synthase to mimic mitochondrial respiration were performed across a range of F26BP concentrations (2×10^{-9} to 5×10^{-4} M) while varying the ratio of V_{max} of glycolysis and respiration: (A) 8:2, (B) 5:5, and (C) 2:8. The glycolytic ATP production rate is represented by PKM2 flux (pink), respiration rate by ATP synthase flux (black), and ATP consumption rate by ATPase flux (teal). The sum of V_{max} of glycolysis and respiration was kept constant. ATP consumption (ATPase Vmax) was set to 20% of total ATP supply capacity and held constant across all conditions. The light blue shaded region indicates the physiological F26BP concentration range (10^{-8} to 10^{-4} M).

bioRxiv preprint doi: https://doi.org/10.64898/2026.01.31.703051; this version posted January 31, 2026. The copyright holder for this preprint (which was not certified by peer review) is the author/funder, who has granted bioRxiv a license to display the preprint in perpetuity. It is made available under aCC-BY 4.0 International license.

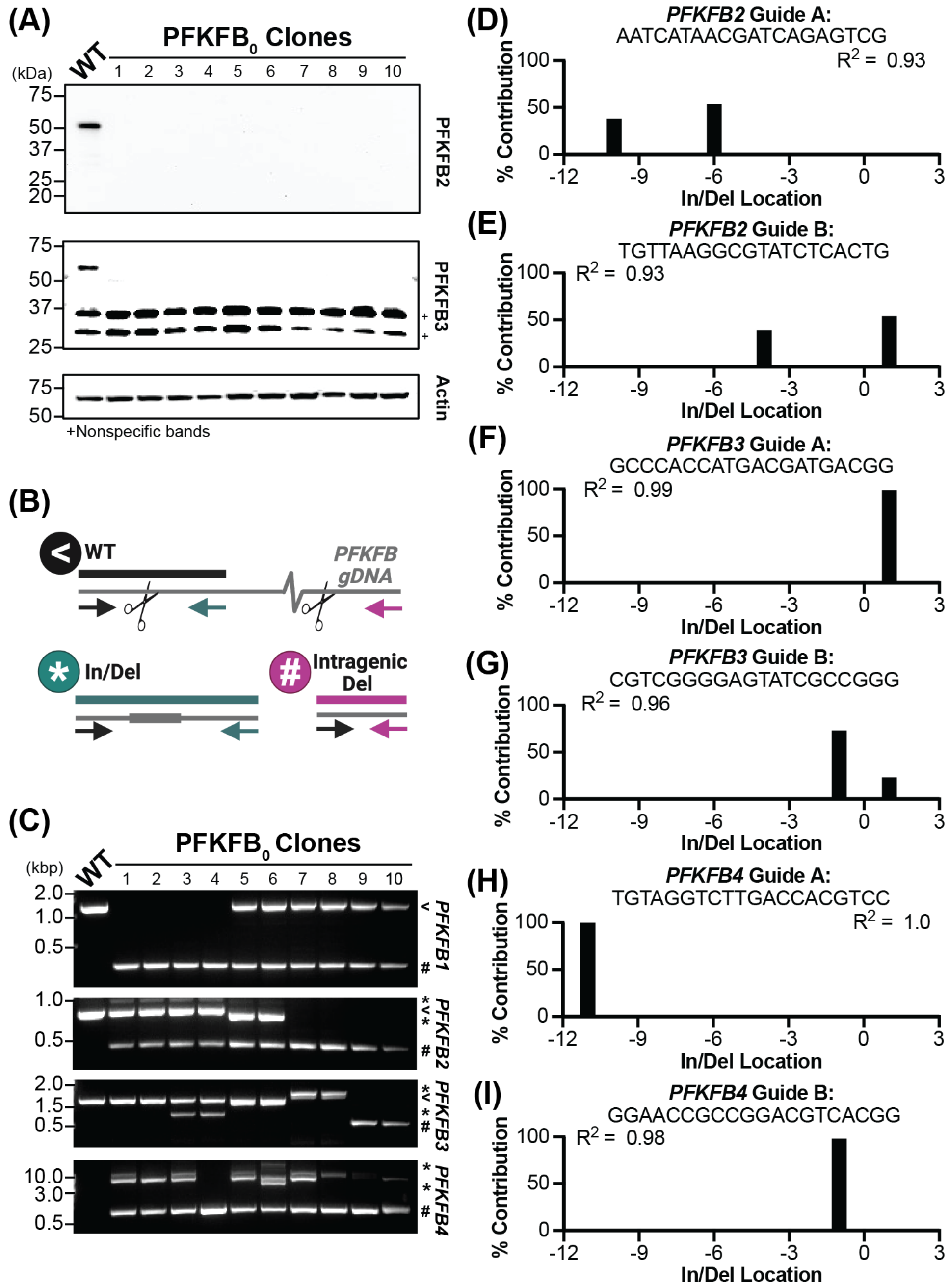

Figure S3. Generation and validation of clonal PFKFB0 knock outs. (A) Western blot showing endogenous PFKFB2 and PFKFB3 expression in HeLa (wildtype, WT) and 10 genetically independent PFKFB0 clones. PFKFB0 clone 1 was utilized for further cell line derivation and investigations throughout this study. Representative gel from one of two independent experiments. (B) Schematic of CRISPR/Cas9 gRNA (scissor) and primer design (arrows), which generated and evaluated insertion/deletion (in/del) and intragenic deletions in PFKFB genes, respectively. (C) PCR amplification of *PFKFB1, PFKFB2, PFKFB3,* and *PFKFB4* genes within WT and 10 genetically distinct PFKFB0 clones. Resolved PCR products are labeled in accordance with B schematic: WT (<), local in/del at amplified cut site (*), and severe intragenic deletion (#). (D-I) Monoallelic knock out efficiency of PFKFB0 clone 1 as evaluated by EditCo's Inference of CRISPR Edits (ICE)[58,59]. Estimates the contribution of insertion/deletions at gRNA targeting: (D) site A within *PFKFB2,* (E) site B within *PFKFB2,* (F) site A within *PFKFB3,* (G) site B within *PFKFB3,* (H) site A within *PFKFB4,* and (I) site B within *PFKFB4.* R^2 denotes Pearson correlation coefficient, indicating how well the ICE model fits with the Sanger Sequencing data.

bioRxiv preprint doi: https://doi.org/10.64898/2026.01.31.703051; this version posted January 31, 2026. The copyright holder for this preprint (which was not certified by peer review) is the author/funder, who has granted bioRxiv a license to display the preprint in perpetuity. It is made available under aCC-BY 4.0 International license.

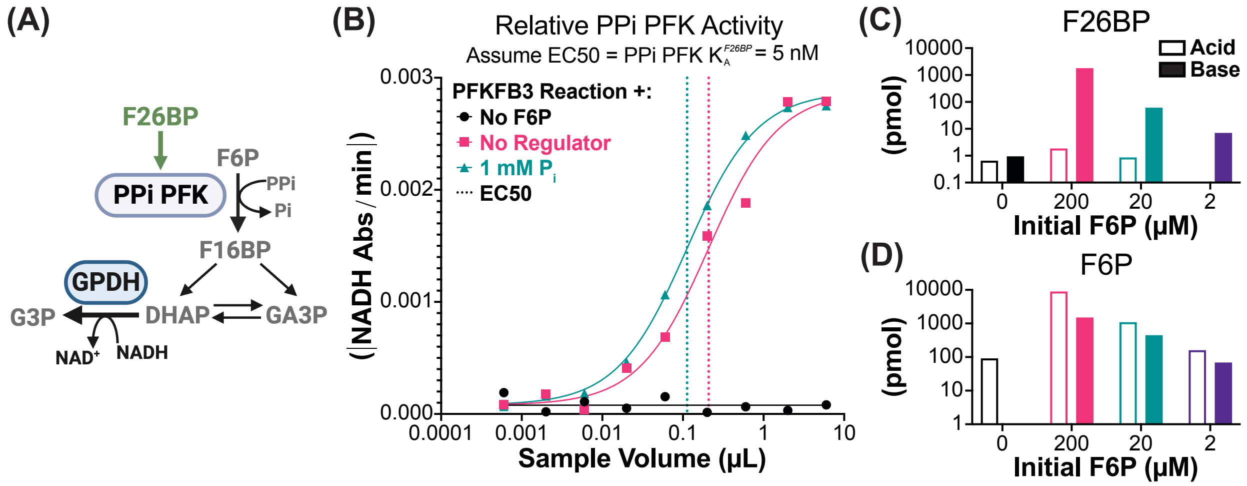

Figure S4. F26BP quantification method. (A) Schematic of the potato pyrophosphate-dependent 6-phosphofructokinase (PP_i PFK) coupled enzyme assay previously described[62]. Notable intermediates (grey text), enzymes (grey ovals), and allosteric regulators (green text) are highlighted. Coupled enzyme reaction terminates with glycerol-3-phosphate dehydrogenase (GPDH), which allows for continuous monitoring by NADH consumption (black text). (B) Representative PP_i PFK EC_{50} curve (solid line) from PFKFB3 reaction serial dilution series. PFKFB3 reaction conditions included: no F6P (black, negative control), no regulator (pink), and the addition of 1 mM P_i (teal, positive control). PP_i PFK EC_{50} (dashed line) was equated to 5 nM F26BP and utilized to calculate F26BP in sample[62]. (C-D) Validating F26BP quantification by quenching PFKFB3 reaction with either acid (outline; F26BP-destabilizing negative control) or base (solid; F26BP-stabilizing positive control). PFKFB3 reaction was incubated with 3 mM ATP and 0 µM (black), 200 µM (pink), 20 µM (teal), or 2 µM (purple) F6P. Measurement from one independent experiment. (C) F26BP content estimated via PP_i PFK activity assay. (D) F6P content quantified via commercial F6P fluorometric kit. In cross comparing F6P utilized to F26BP produced, differences between the acid and base quenching methods correlated with the amount of F26BP stabilized.

bioRxiv preprint doi: https://doi.org/10.64898/2026.01.31.703051; this version posted January 31, 2026. The copyright holder for this preprint (which was not certified by peer review) is the author/funder, who has granted bioRxiv a license to display the preprint in perpetuity. It is made available under aCC-BY 4.0 International license.

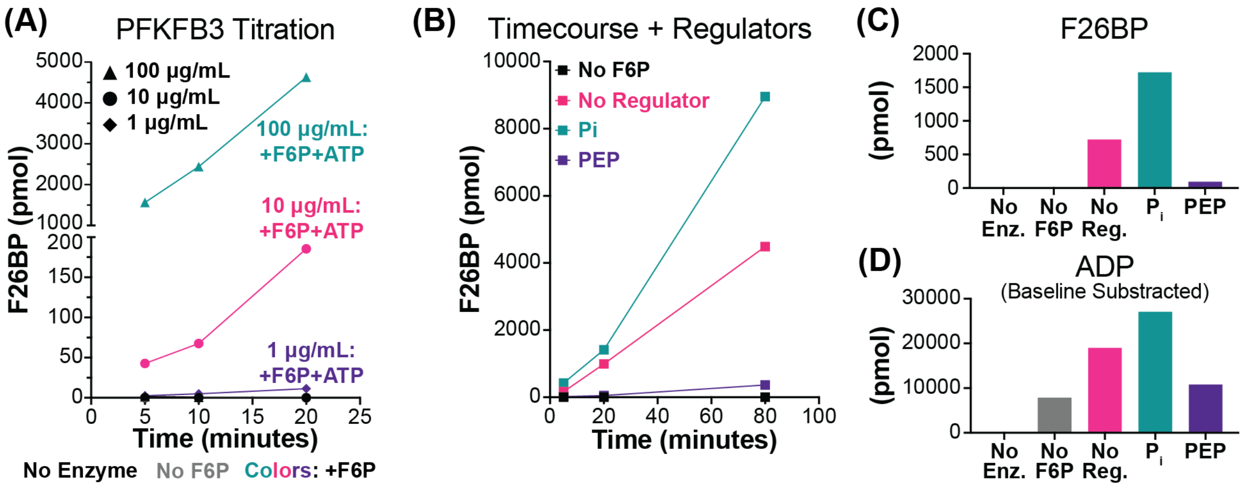

Figure S5. Validating recombinant PFKFB3 activity. (A) Time course with varying PFKFB3 titrations in the presence of 3 mM ATP and 0.2 mM F6P. 10 µg/mL (triangles), 10 µg/mL (circles), 1 µg/mL (diamonds), or 0 µg/mL (black, all shapes) PFKFB3 was incubated with (colors: teal - 100 µg/mL, pink - 10 µg/mL, purple - 1 µg/mL) or without F6P (grey, negative control). (B) PFKFB3 (40 µg/mL) time course in the presence or absence of known allosteric regulators. Reaction conditions included: no F6P (black, negative control), no regulator (pink), the addition of 1 mM P_i (teal, allosteric activator), and the addition of 1 mM PEP (purple, allosteric inhibitor). (C-D) Detection of PFKFB3 (100 µg/mL) kinase activity by incubating or 0 µg/mL (black; No Enz.) PFKFB3 with conditions described for 20 minutes. (C) Estimated F26BP content compared to (D) base-line subtracted ADP content. In cross-comparing the products of the PFKFB3 kinase domain, the F26BP and ADP quantities correlated across the different regulatory conditions.
